# Integrated Analysis of Differential miRNA and mRNA Expression Profiles in Human Radioresistant and Radiosensitive Nasopharyngeal Carcinoma Cells

**DOI:** 10.1371/journal.pone.0087767

**Published:** 2014-01-31

**Authors:** Xin-Hui Li, Jia-Quan Qu, Hong Yi, Peng-Fei Zhang, Hong-Mei Yi, Xun-Xun Wan, Qiu-Yan He, Xu Ye, Li Yuan, Jing-Feng Zhu, Jiao-Yang Li, Zhi-Qiang Xiao

**Affiliations:** 1 Key Laboratory of Cancer Proteomics of Chinese Ministry of Health, Xiangya Hospital, Central South University, Changsha, Hunan, China; 2 Department of Pathology, Medical College of Jishou University, Jishou, Hunan, China; Central South University, China

## Abstract

**Background:**

The purpose of this study was to identify miRNAs and genes involved in nasopharyngeal carcinoma (NPC) radioresistance, and explore the underlying mechanisms in the development of radioresistance.

**Methods:**

We used microarrays to compare the differences of both miRNA and mRNA expression profiles in the radioresistant NPC CNE2-IR and radiosensitive NPC CNE2 cells, applied qRT-PCR to confirm the reliability of microarray data, adopted databases prediction and anticorrelated analysis of miRNA and mRNA expression to identify the miRNA target genes, and employed bioinformatics tools to examine the functions and pathways in which miRNA target genes are involved, and construct a miRNA-target gene regulatory network. We further investigated the roles of miRNA-23a and its target gene IL-8 in the NPC radioresistance.

**Results:**

The main findings were fourfold: (1) fifteen differential miRNAs and 372 differential mRNAs were identified, and the reliability of microarray data was validated for randomly selected eight miRNAs and nine genes; (2) 174 miRNA target were identified, and most of their functions and regulating pathways were related to tumor therapeutic resistance; (3) a posttranscriptional regulatory network including 375 miRNA-target gene pairs was constructed, in which the ten genes were coregulated by the six miRNAs; (4) IL-8 was a direct target of miRNA-23a, the expression levels of IL-8 were elevated in the radioresistant NPC tissues and showed inverse correlation with miRNA-23a expression, and genetic upregulation of miRNA-23a and antibody neutralization of secretory IL-8 could reduce NPC cells radioresistance.

**Conclusions:**

We identified fifteen differential miRNAs and 372 differential mRNAs in the radioresistant NPC cells, constructed a posttranscriptional regulatory network including 375 miRNA-target gene pairs, discovered the ten target genes coregulated by the six miRNAs, and validated that downregulated miRNA-23a was involved in NPC radioresistance through directly targeting IL-8. Our data form a basis for further investigating the mechanisms of NPC radioresistance.

## Introduction

Nasopharyngeal carcinoma (NPC) is an endemic disease in southern China and Southeast Asia, and tends to be more sensitive to ionizing radiation than other head and neck cancers. Thus, the primary treatment for NPC is radiotherapy. Although more accurate tumor localization by computed tomography and better radiotherapy techniques have contributed to the improvement in the local control of NPC, a major impediment to achieve long-term survival is radioresistance [1]. Most of the NPC patients suffer from local recurrence and distant metastasis within 1.5 years after radiotherapy due to radioresistance [2]. Hence, understanding the mechanisms of NPC radioresistance is important for developing the personalized therapy and improving the patient prognosis.

Previous studies have identified some proteins that are associated with NPC radioresistance, such as EB virus-encoded latent membrane protein 1 (LMP1) [3], αV integrin [4], Etk [5], EGFR [6], metallothionein [7], p21 [8], gp96 and GDF15 [9]. In our previous study, a radioresistant cell line (CNE2-IR) derived from poorly differentiated NPC cell line CNE2 was established, and comparative proteomic analysis of CNE2-IR and control CNE2 cells identified the four NPC radioresistance-related proteins [10]. Although these proteins are believed to play a role in the NPC radioresistance, our understanding of NPC radioresistance at a molecular level is limited.

Gene expression regulation through mechanisms that involve microRNAs (miRNAs) has attracted much attention during recent years. miRNA is an important class of small non-coding RNAs that can regulate the expression of protein-coding genes through targeting mRNA degradation and inhibiting mRNA translation. Abnormally expressed miRNAs have been identified as oncogenes or tumor suppressors in the human cancers [11], influencing the pathogenesis and progression of cancers [12]. It has been suggested that miRNAs can modulate tumor radiosensitivity by affecting DNA damage repair, cell cycle checkpoint, apoptosis, and radio-related signal pathways, such as PI3K/Akt, NF-κB, MAPK, TGF-β, Stats and inflammation signaling pathways [13], [14]. Several miRNAs have been shown to be associated with the radioresistance of tumors including NPC. For example, miRNA-205 increased NPC cells radioresistance by directly targeting PTEN [15], miRNA-221 and miRNA-222 regulated gastric carcinoma cells radioresistance by targeting PTEN [16], downregulation of miRNA-210 expression enhanced radiosensitivity in hypoxic human hepatoma cells [17], overexpression of miRNA-421 lead to a pronounced DSB repair defect and clinical hypersensitivity in SKX squamous cell carcinoma [18], silencing of miRNA-21 increased radiosensitivity through inhibiting a PI3K/AKT pathway and enhancing autophagy in malignant glioma cells [19], and upregulation of NF-κB-dependent miRNA-125b promoted cell survival by targeting p38α upon ultraviolet radiation [20].

Various genome-wide miRNA expression profiling studies using microarray-based approaches have provided us with abundant information on the phenotypic characteristics of cancers [21]-[23]. Distinct patterns of miRNA expression and special miRNA signatures were found to be associated with the clinical and pathological characteristics of NPC and the patient outcome [24]–[28]. Nevertheless, few miRNA expression profiling studies have been focused on the tumor radioresistance [29]–[31]. To our knowledge, there has been no report on miRNA expression profiling study of NPC radioresistance.

In this study, we for the first time compared the differences of both miRNA and mRNA expressional profiles in the radioresistant NPC CNE2-IR and radiosensitive CNE2 cells using microarrays, identified the differential miRNA target genes by databases prediction and inverse correlation analysis of miRNA and mRNA expressions, and adopted bioinformatics to analyze the functions and pathways in which the miRNA target genes are involved, and construct a miRNA-target gene regulatory network. We further investigated the roles of downregulated miRNA-23a and its target gene IL-8 in the NPC radioresistance. Our data provide an important contribution to future investigations aimed at elucidating the mechanisms of NPC radioresistance.

## Materials and Methods

### Cell Lines and Tissue samples

Radioresistant NPC cell line CNE2-IR and its control cell line CNE2 were previously established by us [10]. CNE2-IR was derived from poorly differentiated NPC cell line CNE2 by treating the cells with four rounds of sublethal ionizing radiation. CEN-2 cells, used as a control, were treated with the same procedure except sham irradiated [10]. The cells were cultured with DMEM medium supplemented with 10% FCS (Invitrogen, USA) and 1% antibiotics (Invitrogen) in an incubator at 37°C with humidified 5% CO_2_.

The sixty formalin-fixed and paraffin-embedded archival NPC tissue specimens, comprising thirty radioresistant and thirty radiosensitive ones, were obtained from Xiangya Hospital of Central South University (Changsha, China) between January 2007 and June 2009, and used for immunohistochemical staining of IL-8 and qRT-PCR analysis of miRNA-23a. We also acquired ten paraffin-embedded non-cancer nasopharyngitis tissues samples from patients who were originally suspected to have NPC from Xiangya Hospital of Central South University in the same period. NPC and non-cancer nasopharyngitis tissue biopsies were obtained from the patients at the time of diagnosis before any therapy with the written informed consent, and approved by the ethics committee of Xiangya Hospital. Radioresistant and radiosensitive NPC patients were defined according to our previous criteria [10], and the clinicopathological parameters of NPC tissues are shown in Table S1.

### Total RNA Extraction

Total RNA was extracted from CNE2-IR and CNE2 cells with Trizol reagent (Invitrogen) according to manufacturer's protocol. Total RNA was isolated from the formalin-fixed and paraffin-embedded NPC tissues using RecoverAll™ Total Nucleic Acid Isolation Kit (Ambion, USA) according to the manufacturer's protocol. The concentration and integrity of RNA were evaluated using Agilent 2100 Bioanalyzer (Agilent Technologies, USA). Only total RNA samples with RNA integrity number (RIN) ≥7 were used for microarray analyses of miRNA and mRNA.

### miRNA Microarray Assay

Microarray assays of CNE2-IR and CNE2 cells miRNAs were outsourced to CapitalBio Corporation (Beijing, China). To enrich global miRNA, total RNA extract was purified using mirVana™ miRNA Isolation Kit (Ambion, USA), and then labeled and hybridized. Briefly, 100 ng of miRNA was labeled using the Agilent miRNA Complete Labeling and Hybridization Kit (Agilent Technologies, USA) according to the manufacturer's instructions. The labeled RNA was hybridized to the Agilent human miRNA mcroarray which contains probes for 1205 human miRNAs and 144 human viral miRNAs from the Sanger database (Version 16.0) according to the manufacturer's instructions. Arrays were scanned with the Agilent's scanner, and the raw data were normalized and analyzed by GeneSpring GX software version 7.3 (Agilent Technologies). The GeneSpring software generated an average value for each miRNA from the repeated probes. Microarray assay was performed in triplicates, utilizing three independent sets of RNA preparations. miRNA signal intensities were log2 transformed, and analyzed for differentially expressed miRNAs by using the significance analysis of microarrays (SAM, version 3.01), and then the p-values of the t-test were calculated. Differentially detected miRNA signals with ≥1.5 fold-change and the *P*<0.05 were considered significant. Differential miRNAs detected in the triplicate experiments were selected for further analysis. Unsupervised Hierarchical Clustering was performed for the differentially expressed miRNAs with *P*<0.01 using Cluster 3.0 and Java TreeView-1.1.6-win.

### Gene Expression Microarray Assay

Gene expression microarray analyses of CNE2-IR and CNE2 mRNAs were outsourced to CapitalBio Corporation. Affymetrix's Genechip IVT Express Kit was used for cDNA synthesis and in vitro transcription. The gene expression microarray assay was performed using 5 µg of total RNA on an Affymetrix human genome U133 plus 2.0 microarray which analyzes the expression levels of 47,000 transcripts and variants, including 38500 well-characterized human genes (Affymetrix, USA). All procedures and analyses were performed according to the protocols outlined in detail in the geneChip@ expression analysis technical manual (Affymetrix, http://www.affymetrix.com/support/downloads/manuals/expression_analysis_technical_manual.pdf). Microarray assay was performed in duplicates, utilizing two independent sets of RNA preparations. Expression data were subsequently background corrected, normalized, and polished using robust multichip average (RMA) as previously described [32]. mRNA signal intensities were log2 transformed, and analyzed for differentially expressed mRNAs by using the SAM (version 3.01), and the p-values of the t-test were calculated. Differentially detected mRNA signals with ≥2.0 fold-change and the *P*<0.01 were considered significant. Unsupervised Hierarchical Clustering was performed for the differentially expressed mRNAs with *P*<0.01 using Cluster 3.0 and Java TreeView-1.1.6-win.

### Data Availability

The miRNA and mRNA microarray data generated by this study are available in the NCBI Gene Expression Omnibus (GEO) as series accession identifier GSE48503.

### Quantitative Reverse Transcription-PCR

To validate the reliability of microarray data, qRT-PCR was performed to detect the levels of randomly selected nine differential miRNAs and eight differential mRNAs from the differential expression data. For miRNA qRT-PCR, 2 µg of total RNA was reversely transcribed for cDNA using the reverse transcription (RT) kit according to the manufacturer's instructions (Promega, USA), and miRNA specific primers (Bulge-Loop™ miRNA qPCR primers), which were synthesized by RiboBio (Guangzhou, China) and summarized in Table S2. The RT products were amplified by real time-PCR using the miScript SYBR green PCR kit (Qiagen, Germany) according to the manufacturer's instructions, and U6 was used as the internal control. Real time-PCR primers for amplification of miRNAs were designed based on the miRNA sequences provided by the Sanger Center miRNA Registry, which were synthesized by RiboBio and summarized in Table S2.

For mRNA qRT-PCR, 2 µg of total RNA was reversely transcribed for cDNA using the RT kit according to the manufacturer's protocol and Oligo dT primer (Promega) according to the manufacturer's protocol. The RT products were amplified by real-time PCR using QuantiFast SYBR green PCR kit (Qiagen) according to the manufacturer's instructions, and GAPDH was used as the internal control. Real-time PCR primers for amplification of mRNAs were designed by primer premier 5.0 and summarized in Table S3.

All qRT-PCR reactions were performed in triplicates on the ABI Gene Amp PCR System 9700 (ABI, USA), and the products were quantitated using 2^−ΔΔCt^ method against the GAPDH or U6 for normalization.

### Identification of miRNA Target Genes by Databases Prediction and Inverse Correlation Analysis of miRNA and mRNA Expressions

Putative target genes of differentially expressed miRNAs were predicted by five databases with pure algorithm prediction (DIANAmT, miRanda, miRDB, PICTAR5, and Targetscan) and three databases with experiment validation (mirWalk, miRTarBase, and TarBase), respectively. The miRNA target genes recorded by ≥ two databases with pure algorithm prediction or ≥ one database with experiment validation, were selected to compare with the expression profile data. As miRNAs tend to downregulate target mRNAs, the expression of a genuine target mRNA is expected to be anticorrelated with miRNA expression [25], [33], [34]. Therefore, we used inverse correlation analysis of miRNA and mRNA expression to identify more genuine miRNA target genes as previously described [25], [33], [34]. The target genes, which were differentially expressed genes in mRNA microarray assays and anticorrelated with miRNA expression, were selected and subjected to further investigation.

### Analyses of Gene Ontology and KEGG Pathways of miRNA Target Genes

To understand the functions of miRNA target genes, we performed the ontology classification of the target genes based on gene annotation and summary information available through DAVID. KEGG pathway analysis shared by the target genes was also performed using Cytoscape V2.6.3 (http://cytoscape.org/) with the ClueGo plug-in. Correlation is significantly enriched in cases where the corrected p-value was <0.01 with q*-*value of false discovery rate (FDR) <0.01.

### Construction of a miRNA-Target Gene Regulatory Network

The posttranscriptional regulatory network of miRNAs and genes is defined as a directed and bipartite graph in which expressions of miRNA-target gene interacting pairs are anticorrelated. The differentially expressed miRNAs of interest and differentially expressed genes of interest were determined by pathways extracted from KEGG as primary nodes-networks. The networks were drawn using Cytoscape V2.6.3, and the functional enrichment analysis was performed using Database for Annotation, Visualization and Integrated Discovery 2008 Tool (http://david.abcc.ncifcrf.gov/).

### Dual Luciferase Reporter Assay

To test whether miRNA-23a can specifically target IL-8, a dual-luciferase reporter with the 3′UTR of IL-8 (Catalog#, HmiT009678-MT01; GeneCopoeia, USA) and miRNA-203 mimic or mimic control (RiboBio) were cotransfected into the radioresistant NPC CNE2-IR cells using Lipofectamine 2000 as previously described [35]. A dual-luciferase reporter without the 3′UTR of IL-8, pEZX-MT01 (Catalog#, CmiT000001-MT01; GeneCopoeia), was used as a control. Cells were harvested 48 h after transfection, and both firefly luciferase and renilla luciferase activities were measured with the Dual-Luciferase Reporter Assay System (Promeaga) according to the manufacturer's protocol. Statistical significance of the differences in luciferase activity was determined by Student's t test.

### Analysis of miRNA-23a and IL-8 Expression in the NPC Tissues with Different Radiosensitivity

The expression levels of IL-8 in the radioresitant and radiosensitive NPC tissues and nasopharyngitis tissues were detected by immunohistochemical staining using anti-human IL-8 antibody (Abcom, ab18672; 1∶2000 dilution), and the expression levels of IL-8 were evaluated as previously described by us [10]. The expression levels of miRNA-23a in the same NPC tissue samples were detected by qRT-PCR as above described. Significant inverse correlation of miRNA-23a and IL-8 expression was determined by Pearson's correlation analysis.

### Analysis of the Role of miRNA-23a and IL-8 in NPC Radioresistance

To determine the effects of miRNA-23 downregulation on NPC radioresistance, miRNA-23a mimic and mimic control (RiboBio) were transfected into the CNE2-IR cells using riboFect™ CP transfection kit (RiboBio) according to manufacturer's instructions, respectively. 48h after transfection, the expression level of IL-8 was detected by Western blot, and radioresistance of the transfected cells was measured by a clonogenic survival assay and Hoechst 33258 staining of apoptotic cells as previously described by us [10], [36]. To determine the effects of IL-8 upregulation on NPC radioresistance, CNE2-IR cells were cultured with DMEM medium supplemented with 2% FCS and monoclonal mouse anti-human IL-8 antibody (final concentration, 2.5 µg/mL; Abcom, ab18672), and the cells radioresistance was measured by a clonogenic survival assay. Mouse IgG1 isotype nonreactive monoclonal antibody (final concentration, 2.5 µg/mL; R&D, USA) replaced anti-human IL-8 antibody as a negative control.

## Results

### Differentially Expressed miRNAs and mRNAs in the Radioresistant NPC Cells

miRNA expression profiling analysis was carried out for NPC CNE2-IR and CNE2 cells. After filtering miRNAs that were not expressed in the two cell lines, a total of 277 miRNAs were detected by microarray, fifteen of which were differentially expressed with ≥1.5 fold-change (t-test, *P*<0.05). Among them, four miRNAs were upregulated, and eleven miRNAs were downregulated in the radioresistant CNE2-IR cells as compared with the radiosensitive CNE2 cells (Table 1).

**Table 1:** Differentially expressed miRNAs in the CNE2-IR and CNE2 cells detected by microarray.

miRNA	Mean expression		Fold change	*P*-value
	CNE2-IR	CNE2		
**Up-regulated**				
miR-762	−6.39	−7.72	2.51	0.0034
miR-1202	−7.04	−8.24	2.29	0.0008
miR-193b	−2.58	−3.19	1.53	0.0099
let-7e	−2.70	−3.30	1.52	0.0005
**Down-regulated**				
miR-203	−7.07	−5.33	3.34	0.0170
miR-545	−8.95	−7.97	1.98	0.0489
miR-4291	−4.66	−3.88	1.72	0.0027
miR-183*	−8.73	−7.98	1.68	0.0349
miR-24	0.51	1.24	1.67	0.0003
miR-130a	−3.14	−2.46	1.60	0.0125
miR-660	−6.81	−6.15	1.58	0.0153
miR-31*	−3.41	−2.79	1.54	0.0021
miR-23a	1.12	1.73	1.53	0.0355
miR-30a	−3.59	−2.98	1.53	0.0274
miR-30a*	−6.59	−5.99	1.52	0.0328

mRNA expression profiling analysis was also carried out for NPC CNE2-IR and CNE2 cells. mRNAs with the differential levels ≥2.0 fold-change were regarded as significant. As a result, a total of 372 differentially expressed mRNAs were identified, 290 of which were upregulated, and 82 were downregulated in the radioresistant CNE2-IR cells as compared with the radiosensitive CNE2 cells (Table S4).

Fifteen differentially expressed miRNAs and 372 differentially expressed mRNAs were clustered, respectively. The results showed that the expression patterns of both miRNAs and mRNAs could distinguish radioresistant CNE2-IR cells from radiosensitive CNE2 cells (Figure S1 and S2).

### Validation of Microarray Results by qRT-PCR

**Figure 1:**
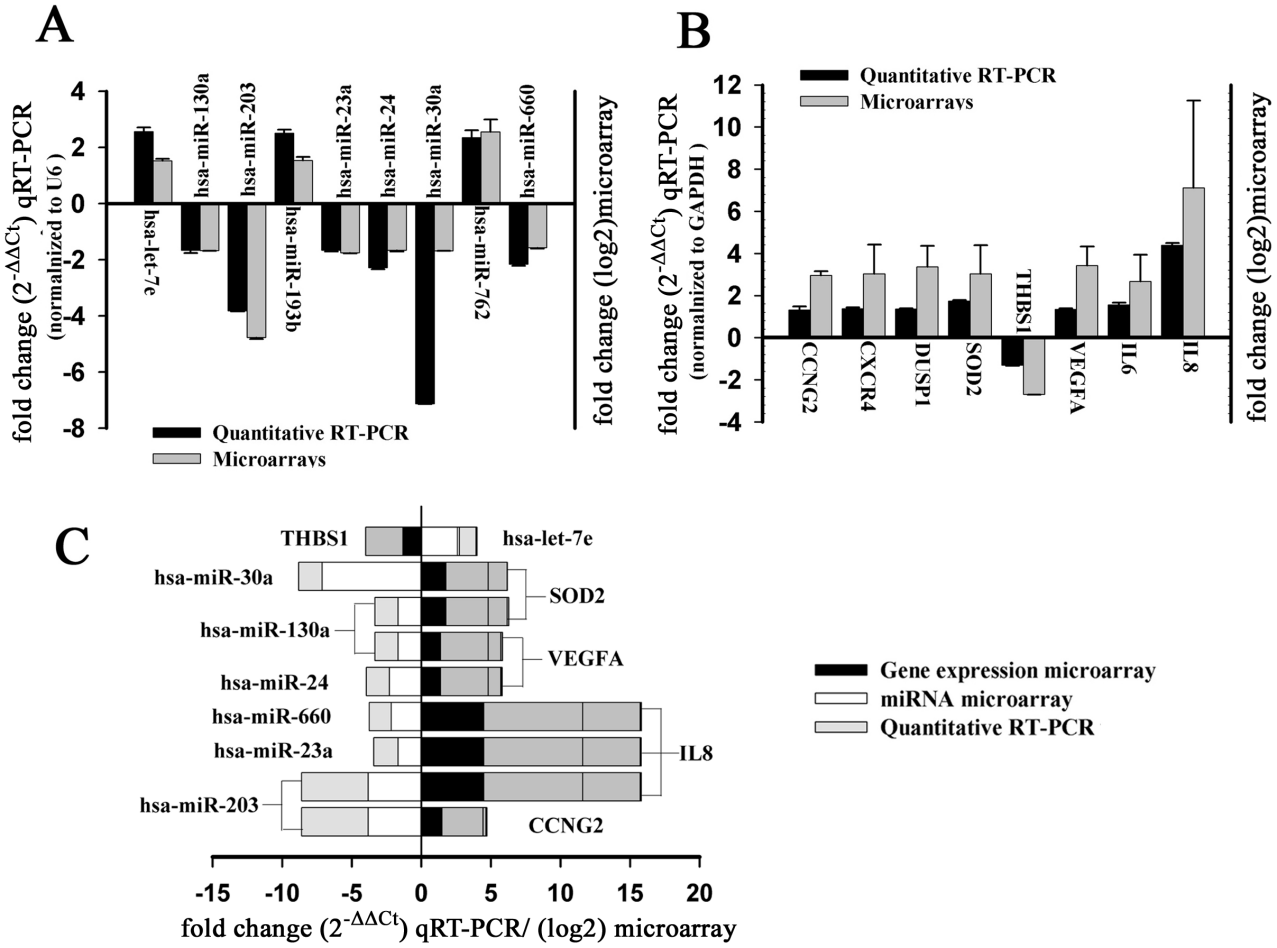
Validation of microarray-based detection of differentially expressed miRNAs and mRNAs in the NPC CNE2-IR and CNE2 cells by qRT-PCR. Nine miRNAs (A) and eight mRNAs (B) selected from micorarry data were detected by qRT-PCR. Fold changes from the microarray were given by log2 values (right y-axis). Fold changes from the qRT-PCR were determined using the 2^−ΔΔCt^ method and normalized to the endogenous control GAPDH or U6 (left y-axis). Error bars represent the standard deviation of the mean (SD). Importantly, the fold changes (y-axis) cannot be directly compared between assays due to differences in calculation methods, but the general trend of upregulation and downregulation can be compared. (C) The nine miRNA-target gene pairs with an inverse correlation of expression identified by microarray analysis and validated by qRT-PCR.

To validate the reliability of microarray data, qRT-PCR was perfromed to detect the levels of randomly selected nine miRNAs and eight mRNAs from the differential expression data in the CNE2-IR and CNE2 cells. The results showed that miRNA-24, miRNA-660, miRNA-203, miRNA-130a, miRNA-30a, and miRNA-23a were significantly decreased, whereas let-7e, miRNA-193b, and miRNA-762 significantly increased in the CNE2-IR cells as compared with CNE2 cells, which was consistent with the results of miRNA microarray analyses (Figure 1A). qRT-PCR also showed that THBS1 were significantly decreased, whereas CCNG2, CXCR4, DUSP1, SOD-2, VEGFA, IL-6, and IL-8 significantly increased in the CNE2-IR cells as compared with CNE2 cells, which was consistent with the results of mRNA microarray analyses (Figure 1B).

### Identification of miRNA Target Genes in the Radioresistant NPC Cells

Putative target genes of fifteen differentially expressed miRNA in the radioresistant CNE2-IR cells were predicted applying the eight databases as described in the Materials and Methods. The target genes recorded by ≥ two databases with pure algorithm prediction or ≥ one database with experiment validation were 20275. To get more genuine target genes, we compared the expression profiles data to identify the target genes anticorrelated with miRNA expression. As a result, 174 target genes were identified, which were differentially expressed genes in the mRNA microarray assays and anticorrelated with the expressions of eleven differentially expressed miRNAs in miRNA microarray assays (Table 2). Furthermore, qRT-PCR analyses confirmed the anticorrelated expressions of nine miRNA-target gene pairs in microarray analyses (Figure 1C).

**Table 2:** The 174 miRNA target genes anticorrelated with the expressions of eleven differentailly expression miRNAs.

miRNA microarray		Gene expression microarray	
miRNA	Expression	Target gene	Expression
miR-193b	Up	CALB1, CCDC88C, MARCKSL1, PSMD11, SMC4	Down
let-7e	Up	ADRB1, GDAP1, HMGA2, KRT5, MARCKSL1, NNT, SIX2, THBS1, ZNF823	Down
miR-203	Down	AFAP1, ANKRD37, ARRDC3, AVPI1, BACH1, BCAR3, BMP2, C1RL, C8orf4, CBS, CCNG2, CDKN2B, COL4A4, CPEB4, CPM, CPS1, CREB5, CREBL2, CSGALNACT1, CXCL2, DLX2, DUSP5, EGR1, EMP1, FAM105A, FAM129A, FAM84B, FOS, FOSL2, GABARAPL1, HPGD, IL6, IL8, IRAK2, IRS2, JAK1, JUN, KLHL24, LIMCH1, MBNL2, MTUS1, MXI1, NEBL, NEDD9, NFIL3, PCSK1, PDE4D, PELI2, PHLDA1, PHLDB2, PPARGC1A, PPP1R3B, RNASE4, SLC1A4, SLC2A3, SLC6A14, SMAD2, SMAD9, SNAI2, SOCS6, STEAP4, TFPI, TNC, TRIM2, WDR32, ZNF292	Up
miR-545	Down	ABCG1, ACSL5, AFAP1, ARRDC3, BMP2, C11orf75, C16orf75, C8orf4, CASP9, CDKN2B, CPEB4, CPM, CREB5, CREBL2, DUSP1, EGLN3, EGR1, EMP1, FOSL2, FZD10, GABARAPL1, GALNT6, GTPBP2, H1F0, HERPUD1, HMHA1, IGFBP3, IRAK2, JAK1, LIMCH1, LMO4, METTL7A, MXI1, NEBL, NEDD9, NRIP1, P4HA1, PCSK1, PDE4D, PELI2, PHLDA1, PHLDB2, PSAT1, RHOU, RNASE4, RPS6KA2, SESN2, SLC1A4, SMAD2, SNAI2, SOCS6, SPINK6, STC1, STC2, STEAP4, TFPI, TMTC1	Up
miR-183*	Down	ARRDC3, BNIP3L, EGR1, NRIP1, SOCS6, TRIM2	Up
miR-24	Down	ABCG1, ADM, ADSSL1, AFAP1, AGR2, BACH1, BLVRB, BNIP3L, CBS, CDKN1B, CPM, CPS1, CREB5, CREBL2, DLX2, DUSP16, EMP1, ENO2, FOS, FOSL2, FUT1, FZD10, GALNT6, GDPD1, INHBE, IRAK2, JUN, KLHL24, LAMB3, LOX, MMP10, MXI1, NDRG1, NEDD9, PADI1, PCTK2, PDE4D, PDK1, PFKFB4, PHLDA1, PLOD2, PRDM1, PSCA, RAB40C, RNF24, SLC6A14, SMAD2, SOCS6, STC2, TFPI, TMTC1, TNFAIP2, TRIB3, TSC22D3, ULK1, VEGFA, WDR32	Up
miR-130a	Down	ACSL5, ANG, ARRDC3, C8orf4, CASP9, CDKN2B, COL4A4, CPEB4, CREB5, EGLN3, ERRFI1, FAM129A, FOS, FOSL2, GABARAPL1, GADD45A, GADD45B, MAFF, MMP10, NEBL, NOG, PDE4D, PDK1, PLSCR4, PPARGC1A, RBCK1, RPS6KA2, SDC4, SESN2, SLC2A1, SLC2A3, SMAD2, SOCS6, SOD2, STC1, STEAP4, STXBP1, TMTC1, TRIM2, VEGFA, WDR32	Up
miR-660	Down	ABCA12, ADM, AFAP1, ARRDC3, C9orf150, CLIC5, FUT1, IL8, INHBE, IRS2, LIMCH1, LOX, METTL7A, MTUS1, PBLD, PPARGC1A, SDC4, SEMA6D, SLC1A4, STEAP4, TMEM139, WDR32	Up
miR-31*	Down	CDKN2B, EMP1, FOS, HBEGF, IL6, IL8, SOD2	Up
miR-23a	Down	ADM, ARRDC3, BNIP3L, C11orf75, CDKN2B, COL4A4, CPEB4, CPM, CPS1, CREB5, CXCL2, DUSP5, ENO2, FAM129A, FUT1, GBP1, GBP2, HPGD, IL8, INHBE, INSIG2, IRS2, JAK1, KLHL24, NEDD9, NTN4, PCTK2, PFKFB4, PHLDA1, PLEKHA2, PNMA2, PPARGC1A, PRDM1, RNASE4, RSAD2, SAMD11, SEMA6D, SERTAD4, SESN2, SLC16A6, SLC2A1, SLC6A14, SMAD2, SOCS6, TNFAIP2, TNFAIP3, TNFAIP6, TRIB1, TRIM2, ZNF292	Up
miR-30a	Down	ABCA12, BACH1, BNIP3L, C8orf4, CAMK2N1, CARS, CBS, CPEB4, CSGALNACT1, CTH, ERRFI1, FAM105A, FZD10, GABARAPL1, GALNT6, GBP1, INSIG2, IRF2BP2, IRS2, JAK1, JUN, KLHL24, LIMCH1, MAP1LC3B, MBNL2, METTL7A, NEBL, NRIP1, P4HA1, PCTK2, PDE4D, PFKFB4, PHLDB2, PPARGC1A, PPP1R3B, PRDM1, PSAT1, RHOB, RPS6KA2, RSAD2, SEMA6D, SLC1A4, SLC2A3, SMAD2, SOCS6, SOD2, STC1, STEAP4, STXBP1, TMEFF1, TMTC1, TNFAIP2, TRIB3, TRIM31	Up

### Gene Ontology and KEGG Pathways of miRNA Target Genes

The 174 miRNA target genes were formulated into an XML-based input data set to query the GO database. The results showed that 117 GO functions were annotated (data not shown). The most enriched GO terms of the miRNA target genes were oxidation reduction, response to hypoxia, signal transduction, cell-cell signaling, cell cycle arrest, negative regulation of progression through cell cycle, cell cycle and apoptosis, inflammatory response, and immune response etc.(Table 3).

**Table 3:** GO functional annotation of 174 miRNA target genes.

Term	Count	%	*P*-value	FDR
GO:0006355 regulation of transcription, DNA-dependent	19	10.92	1.09E-16	3.28E-16
GO:0045944 positive regulation of transcription from RNA polymerase II promoter	10	5.75	2.60E-16	7.23E-16
GO:0055114 oxidation reduction	10	5.75	2.36E-12	5.41E-12
GO:0007050 cell cycle arrest	6	3.45	2.41E-11	5.00E-11
GO:0007275 development	14	8.05	1.09E-10	2.03E-10
GO:0045786 negative regulation of progression through cell cycle	6	3.45	4.54E-10	7.09E-10
GO:0006469 negative regulation of protein kinase activity	5	2.87	4.93E-10	7.39E-10
GO:0006954 inflammatory response	7	4.02	6.51E-10	9.40E-10
GO:0006350 transcription	13	7.47	2.54E-09	3.09E-09
GO:0008285 negative regulation of cell proliferation	6	3.45	1.37E-08	1.44E-08
GO:0007267 cell-cell signaling	7	4.02	6.00E-08	5.32E-08
GO:0000122 negative regulation of transcription from RNA polymerase II promoter	5	2.87	7.72E-08	6.69E-08
GO:0001666 response to hypoxia	4	2.30	9.47E-08	7.54E-08
GO:0007165 signal transduction	13	7.47	1.29E-07	9.85E-08
GO:0007155 cell adhesion	7	4.02	2.75E-07	1.91E-07
GO:0007049 cell cycle	7	4.02	6.09E-07	4.03E-07
GO:0006915 apoptosis	7	4.02	6.28E-07	4.08E-07
GO:0006955 immune response	6	3.43	1.04E-05	4.28E-06
GO:0030154 cell differentiation	7	4.00	9.73E-06	4.04E-06

The 174 miRNA target genes were also uploaded into KEGG database for pathway enrichment analysis. The results showed that twenty-nine pathways including p53 signaling pathway, TGF-beta signaling pathway, focal adhesion, MAPK signaling pathway, mTOR signaling pathway, cell cycle, cytokine-cytokine receptor interaction, Toll-like receptor signaling pathway, and insulin signaling pathway were statistically enriched (Table S5).

### The Posttranscriptional Regulatory Network of miRNAs and Target Genes

**Figure 2:**
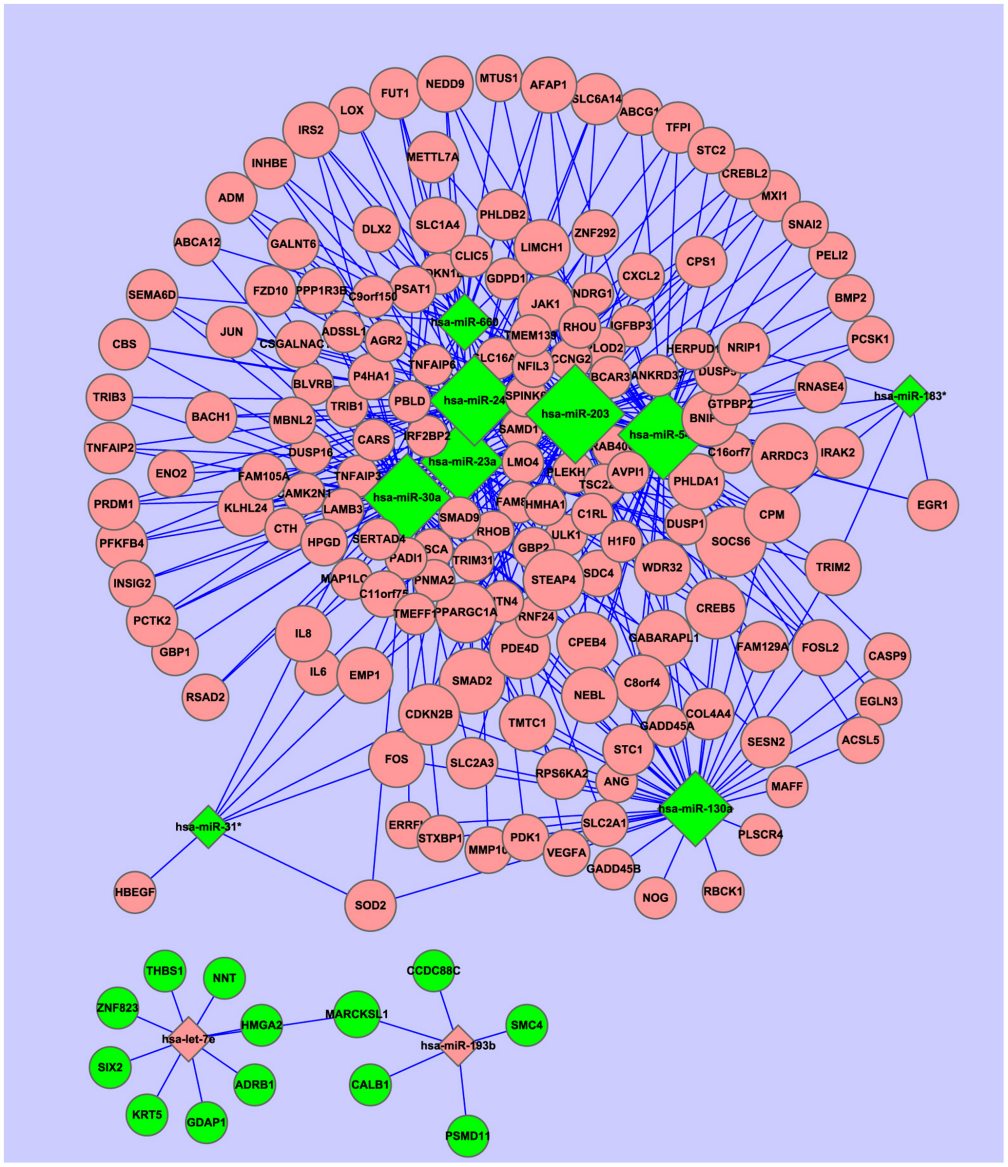
The posttranscriptional regulatory network of miRNAs and target genes in the radioresistant NPC cells. Eleven miRNAs and 174 target genes with an inverse correlation of expression were built into a bipartite network using Cytoscape v2.6. The diamonds and ellipses represent the miRNAs and genes, respectively. The red and green colors represent the relatively high and low expression, respectively. The larger geometric drawing indicates the more miRNAs or genes interacted with it.

The miRNA-target genes regulatory network in the radioresistant NPC cells was constructed using the miRNA-target gene pairs as described in the Materials and Methods. As a result, eleven miRNAs and 174 genes formed 375 miRNA-target gene pairs with an inverse correlation of expression (Table S6). Using the 375 miRNA-target gene pairs, a miRNA-target gene regulatory network was constructed (Figure 2). In this network, ten genes (SOCS6, SMAD2, CDKN2B, PPARGC1A, FOS, FOSL2, IL8, IRS2, JAK1, WDR32) were coregulated by six miRNAs (miRNA-23a, miRNA-24, miRNA-30a, miRNA-545, miRNA-203, miRNA-660) (Figure 2, Table 4).

**Table 4:** Ten genes coregulated by six miRNAs identified by the miRNA-target genes regulatory network.

miRNA	Gene
miR-203	CDKN2B, FOS, FOSL2, IL8, IRS2, JAK1, PARGC1A, SMAD2, SOCS6, WDR32
miR-23a	CDKN2B, IL8, IRS2, JAK1, PPARGC1A, SMAD2, SOCS6
miR-24	FOS, FOSL2, SMAD2, SOCS6, WDR32
miR-30a	IRS2, JAK1, PPARGC1A, SMAD2, SOCS6
miR-545	CDKN2B, FOSL2, JAK1, SMAD2, SOCS6
miR-660	IL8, IRS2, PPARGC1A, WDR32

### Validation of IL-8 as a Target of miRNA-23a in NPC cells

**Figure 3:**
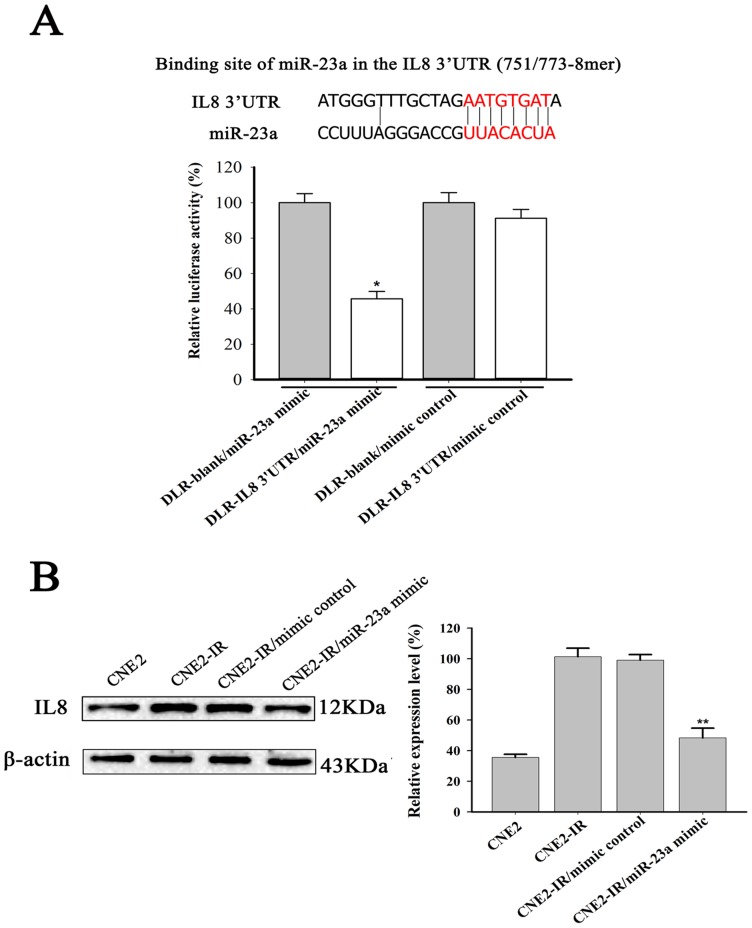
Validation of IL-8 as a target of miRNA-23a. (A) (*top*) Diagrammatic representation of binding site of miRNA-23a in the 3′UTR of IL-8; (*bottom*) miRNA-23a mimic significantly reduced the luciferase activity of a dual luciferase reporter with the 3′UTR of IL-8 compared to the controls. Values are the means ± SD of percent changes over controls after normalization to the Renilla luciferase activity. (B) A representative result of Western blot shows the expression level of IL-8 in the CNE2 and CNE2-IR cells, and CNE2-IR cells transfected with miRNA-23a mimic or mimic control. β-actin was used as an internal control for loading. Three experiments were done; columns, mean; bars, S.D. *P<0.05 and **P<0.01 differ from the controls. DLR-blank, a dual luciferase reporter without the 3′UTR of IL-8; DLR-IL8 3′UTR, a dual luciferase reporter with the 3′UTR of IL-8.

In the miRNA-gene regulatory network of radioresistant NPC cells, IL-8 was cotargeted by the three down-miRNAs (miRNA-203, miRNA-23a and miRNA-660) (Figure 2, Table 4), which was validated by qRT-PCR analysis (Figure 1C). To test whether IL-8 is a direct target of miRNA-23a in NPC cells, a dual luciferase reporter with the 3′UTR of IL-8 or without the 3′UTR of IL-8 was cotransfected with miRNA-23a mimic or mimic control into CNE2-IR cells. CNE2-IR cells cotransfected with miR-23a mimic and a dual luciferase reporter with the 3′UTR of IL-8 exhibited a 55.5% reduced luciferase activity as compared with the cells cotransfected by mimic control and a dual luciferase reporter with the 3′UTR of IL-8, and no significant change of luciferase activity was detected in the cells cotransfected by a dual luciferase reporter without the 3′UTR of IL-8 and miRNA-23a mimic or mimic control (Figure. 3A). In addition, Western blot showed that the expression level of IL-8 in the CNE2-IR was significantly higher than that in the CNE2 cells, and transfection of miRNA-23a into CNE2-IR cells resulted in significant inhibition of IL-8 protein expression as compared with the cells transfected by the mimic control (Figure. 3B). The results demonstrated that IL-8 is a direct target of miRNA-23a in the NPC cells.

### The Expressions of miRNA-23a and IL-8 in the NPC Tissues with Different Radiosensitivity and Their Roles in NPC Radioresistance

**Figure 4:**
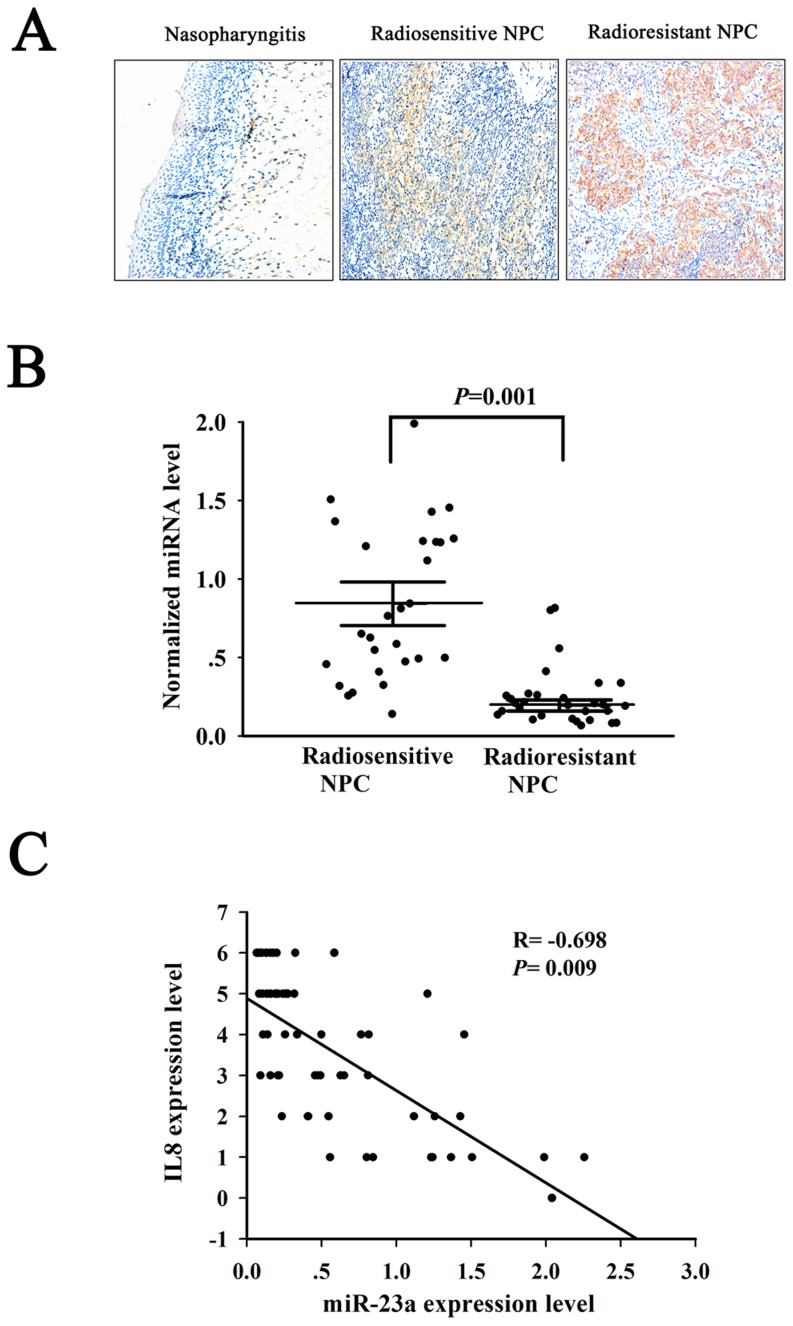
The Expressions of miRNA-23a and IL-8 in the NPC tissues with different radiosensitivity. (A) A representative immunohistochemical result shows no detectable IL-8 expression in the nasopharyngitis tissue, low IL-8 expression in the radiosensitive NPC tissue, and high IL-8 expression in the radioresistant NPC tissue. Original magnification, ×200. (B) Expression levels of miRNA-23a in the radiosensitive and radioresistant NPC tissues. (C) Correlation analysis between IL-8 and miRNA-23a. Pearson's correlation coefficient and P-value for individual analysis are shown in the inserts.

To understand the roles of miRNA-23a and its target gene IL-8 in NPC radioresistance, we first detected the expression of miRNA-23a and IL-8 in the radioresistant and radiosensitive NPC tissues. Immunohistochemistry showed that IL-8 expression was significantly increased in the radioresistant NPC as compared with the radiosensitive NPC, whereas there was no detectable IL-8 expression in the nasopharyngitis (Figure 4A, Table 5). qRT-PCR showed that miRNA-23a expression was significantly decreased in the radioresistant NPC as compared with the radiosensitive NPC (Figure 4B). Furthermore, the expression levels of IL-8 were inverse correlation with miRNA-23a expression (Pearson's correlation coefficient  = −0.698, P<0.01) (Figure 4C). These results indicated that IL-8 might be a target of miRNA-23a in the NPC tissues, and downregulaion of miRNA-203 and upregulation of IL-8 might be involved in the clinical NPC radioresistance.

**Table 5:** The expressions of IL-8 in the radioresistant and radiosensitive NPC tissues detected by immunohistochemistry.

*n*	Expression level (Score)			P-value
	0∼2	3∼4	5∼6	
radiosensitive NPC 30	14	11	5	0.002
radioresistant NPC 30	4	9	17	

Chi square test, *P* = 0.002.

**Figure 5:**
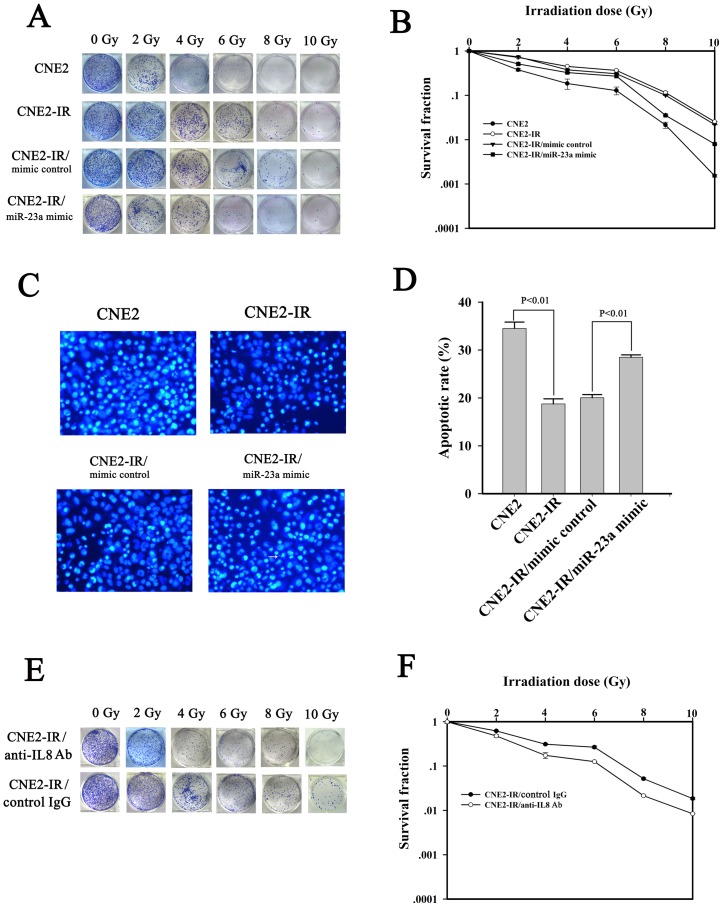
The roles of miRNA-23a and IL-8 in the radioresistance of NPC cells. (A) and (B). A representative clonogenic survival assay shows that transfection of miRNA-23a mimic decreased the radioresistance of NPC CNE2-IR cells. CNE2-IR cells and its transfectants were irradiated with a range of 2-10 Gy radiation doses, and colonies that formed after incubation of 12 d were counted to calculate the survival fractions, and dose survival curve was drawn. (C) Hoechst 33258 staining shows that transfection of miRNA-23a mimic increased the apoptosis of irradiation-induced CNE2-IR cells. CNE2-IR cells and its transfectants were exposed to 6 Gy irradiation, incubated for 48 h, and then assessed for cell apoptosis using the cell-permeable DNA dye Hoechst 33258. (D) A histogram shows the apoptotic rate of CNE2-IR cells and its transfectants 48 h after 6 Gy irradiation. (E) and (F) A representative clonogenic survival assay shows that neutralization of secretory IL-8 using anti-human IL-8 antibody decreased the radioresistance of NPC CNE2-IR cells. CNE2-IR cells were cultured with DMEM medium supplemented with 2% FCS and monoclonal mouse anti-human IL-8 antibody (2.5 µg/mL) or mouse control IgG1 (2.5 µg/mL), and irradiated with a range of 2-10 Gy radiation doses, and colonies that formed after incubation of 12 d were counted to calculate the survival fractions, and dose survival curve was drawn.

To validate the effect of dowregulated miRNA-23a on the NPC radioresistance, miRNA-23a mimic was transfected into CNE2-IR cells, and radiosensitivity of the transfected cells was determined. A clonogenic survival assay showed that transfection of miRNA-23a mimic significantly increased the cells radiosensitivity as compared with transfection of mimic control (Figure 5A and B). Hoechst 33258 staining showed that transfection of miRNA-23a mimic increased irradiation-induced cell apoptosis compared with transfection of mimic control (Figure 5C and D). To validate the effect of upregulated IL-8 on the NPC radioresistance, anti-human IL-8 antibody was added to the medium of CNE2-IR cells to neutralize secretory IL-8, and the cells radiosensitivity was determined. A clonogenic survival assay showed that neutralization of IL-8 significantly increased the cells radiosensitivity as compared with the control mouse IgG1 (Figure 5E and F). These results demonstrated that miRNA-23a downregulation and IL-8 upregulation were involved in NPC cells radioresistance.

## Discussion

In this study, we identified fifteen differentially expressed miRNAs in the radioresistant CNE2-IR cells using microarray. Interestingly, most of them have previously been found to be involved in tumor therapeutic resistance [37]–[44]. miRNA-31 downregulation conferred resistance to radiotherapy and chemotherapy in several types of cancers [37], [38], and downregulation of miRNA-30a [39], miRNA-203 [40], miRNA-183 [41], miRNA-130a [42], miRNA-24 [43] and miRNA-23a [43], and upregulation of miRNA-193b [44] increased tumor cells resistant to chemotherapy. Our results showed that miRNA-23a, miRNA-203, miRNA-31, miRNA-30a, miRNA-183, miRNA-130a, and miRNA-24 were downregulated, and miRNA-193b upregulated in the radioresistant NPC cells, suggesting that deregulation of these miRNAs might be involved in the NPC radioresistance.

As miRNAs exert their roles through degrading target mRNAs or inhibiting target mRNAs translation, thus identification of miRNA target genes is a key step for understanding the biological functions of miRNAs. The computational prediction of miRNA targets currently presents several significant challenges because all of the most widely used databases are characterized by a significant proportion of false-positive interactions [45], [46]. To get more genuine target genes, the putative target genes of fifteen differentially expressed miRNAs predicted by the databases were compared with the expression profiles data to identify the target genes anticorrelated with miRNA expression. As a result, 174 target genes, which were anticorrelated with the expressions of eleven differentially expressed miRNAs, were identified. The other four differentially expressed miRNAs (miRNA-762, miRNA-1202, miRNA-4291 and miRNA-30a*) were not found to have the target genes anticorrelated with their expressions, indicating that they regulated the expression of target genes possibly by translational inhibition.

To estimate the biological functions of differentially expressed miRNAs in NPC radioresistance, we performed GO and KEGG pathway enrichment analysis of the 174 miRNA target genes. The most enriched GO terms of the target genes, such as dysregulation of oxidation reduction, hypoxia, inflammatory, signal transduction, cell cycle and apoptosis, have been reported to be associated with tumor radioresistance [47]–[52], suggesting that the differential miRNAs may be involved in NPC radioresistance through affecting these biological processes. KEGG pathway enrichment analysis showed that twenty-nine pathways were statistically enriched. Among them, p53 signaling pathway, TGF-beta signaling pathway, focal adhesion, MAPK signaling pathway, mTOR signaling pathway, cell cycle, cytokine-cytokine receptor interaction, toll-like receptor signaling pathway, and insulin signaling pathway have been suggested to be involved in tumor therapeutic resistance [13], [50], suggesting that the differential miRNAs may be involved in NPC radioresistance by regulating these pathways.

To identify the putative functional regulatory effects of the differential miRNAs on their targets, we constructed a miRNA-gene regulatory network. In this network, the ten genes were coregulated by the six miRNAs, suggesting that the six miRNAs and ten genes may play important roles in the NPC radioresistance. Previous studies strongly support our views: (1) the three miRNAs (miRNA-23a, miRNA-203 and miRNA-660) target IL-8, a inflammatory factor, not only played an important role in the pathogenesis of NPC [53], but also could activate NF-κB and Stats signaling pathways related to tumor radioresistance [49], [54]; (2) the four miRNAs (miRNA-23a, miRNA-203, miRNA-30a, miRNA-545) target JAK1 is an upstream factor of Stats, and a Stat signaling pathway played an important role in the pathogenesis of NPC [55] and radioresistance of tumors including NPC [14], [54]; (3) the five miRNAs (miRNA-23a, miRNA-203, miRNA-24, miR30a, miRNA-545) target SMAD2 is one member of TGF-β signaling pathway, and activation of this pathway was associated with tumor radioresistance [56], [57]; (4) the five miRNAs (miRNA-23a, miRNA-203, miRNA-30a, miRNA-24, miRNA-545) target SOCS6 is one member of SOCS family, and reciprocal regulation of SOCS1 and SOCS3 enhanced glioblastoma multiforme radioresistance [58]; (5) the two miRNA (miRNA-203, miRNA-24) target FOS, a component of transcriptional factor AP1, was associated with tumor chemoresistance [59], [60].

In the miRNA-target gene regulatory network, IL-8 was cotargeted by the three miRNAs (miRNA-23a, miRNA-203 and miRNA-660). Therefore, we selected one of miRNA-target gene pairs, miRNA-23a and IL-8, for further investigation. A dual-luciferase reporter system assay showed that miRNA-23a could directly bind with the 3′UTR of IL-8 in the radioresistant NPC cells. Furthermore, the expression level of IL-8 in the radioresistant NPC cells was significantly higher than that in the radiosensitive NPC cells, and transfection of miRNA-23a into the radioresistant NPC cells resulted in significant inhibition of IL-8 protein expression. These results demonstrated that IL-8 is a direct target of miRNA-23a in the radioresistant NPC cells.

To understand the effects of miRNA-23a and its target gene IL-8 on NPC radioresistance, we first detected the expression of miRNA-23a and IL-8 in the radioresistant and radiosensitive NPC tissues. The results showed that IL-8 expression was significantly increased, whereas miRNA-23a expression was significantly decreased in the radioresistant NPC tissues as compared with the radiosensitive NPC tissues. Furthermore, the expression levels of IL-8 were inverse correlation with the expression levels of miRNA-23a. These results indicated that IL-8 might also be a target of miRNA-23a in the NPC tissues, and downregulaion of miRNA-203 and upregulation of IL-8 might be involved in the clinical NPC radioresistance. Next, the effect of dowregulated miRNA-23a on the radioresistance of NPC CNE2-IR cells was determined, and both clonogenic survival assay and Hoechst 33258 staining of apoptotic cells showed that transfection of miRNA-23a mimic significantly increased the radiosensitivity of CNE2-IR cells. Finally, the effect of upregulated IL-8 on the radioresistance of NPC CNE2-IR cells was determined, and a clonogenic survival assay showed that neutralization of secretory IL-8 using anti-human IL-8 antibody significantly increased the radiosensitivity of CNE2-IR cells. Taken together, these results demonstrated that miRNA-23a downregulation played an important role in NPC radioresistance through targeting IL-8.

In summary, we identified fifteen differentially expressed miRNAs, 372 differentially expressed mRNAs, and 174 miRNA target genes anticorrelated with miRNA expressions in the radioresistant NPC cells, and constructed a posttranscriptional regulatory network including 375 miRNA-target gene pairs. We for the first time showed that IL-8 was a direct target of miRNA-23a, and upregulated miRNA-23a played an important role in NPC radioresistance by targeting IL-8. Our data are helpful for elucidating the molecular mechanism of NPC radioresistance.

## Supporting Information

Figure S1 **Clustering results of fifteen differentially expressed miRNAs in the CNE2-IR and CNE2 cells.** Unsupervised hierarchical clustering was performed using pearson correlation coefficient and average linkage as distance and linkage metrics, respectively. Samples are well separated into CNE2-IR and CNE2 cells by the differentially expressed miRNAs. Each row represents a miRNA, and each column represents a sample. The red and green colors denote relatively high and low expression, respectively.
(TIF)

Figure S2 **Clustering results of 372 differentially expressed mRNAs in CNE2-IR and CNE2 cells.** Unsupervised hierarchical clustering was performed using pearson correlation coefficient and average linkage as distance and linkage metrics, respectively. Samples are well separated into CNE2-IR and CNE2 cells by the differentially expressed mRNAs. Each row represents a mRNA, and each column represents a sample. The red and green colors denote relatively high and low expression, respectively.
(TIF)

Table S1 **The clinicopathological parameters of the nasopharygeal carcinoma tissue specimens.**
(DOC)

Table S2 **Bulge-Loop^TM^ miRNA qPCR primers item number of miRNAs and U6.**
(XLS)

Table S3 **Gene symbols, primer sequences, and product sizes of selected 8 mRNAs.**
(XLS)

Table S4 **Differentially expressed mRNAs in radioresistantCNE2-IR and radiosensitive CNE-2 cells.**
(XLS)

Table S5 **KEGG pathway annotation of 174 target genes with an inverse correlation expression of 11 miRNAs.**
(XLS)

Table S6 **miRNA-target gene pairs with an inverse correlation of expression.**
(XLS)
