# Supplementary figures and images for: Integrated Analysis of Differential miRNA and mRNA Expression Profiles in Human Radioresistant and Radiosensitive Nasopharyngeal Carcinoma Cells
Source: PLoS One. 2014 Jan 31;9(1):e87767. doi: 10.1371/journal.pone.0087767 (PMC3909230; doi:10.1371/journal.pone.0087767)

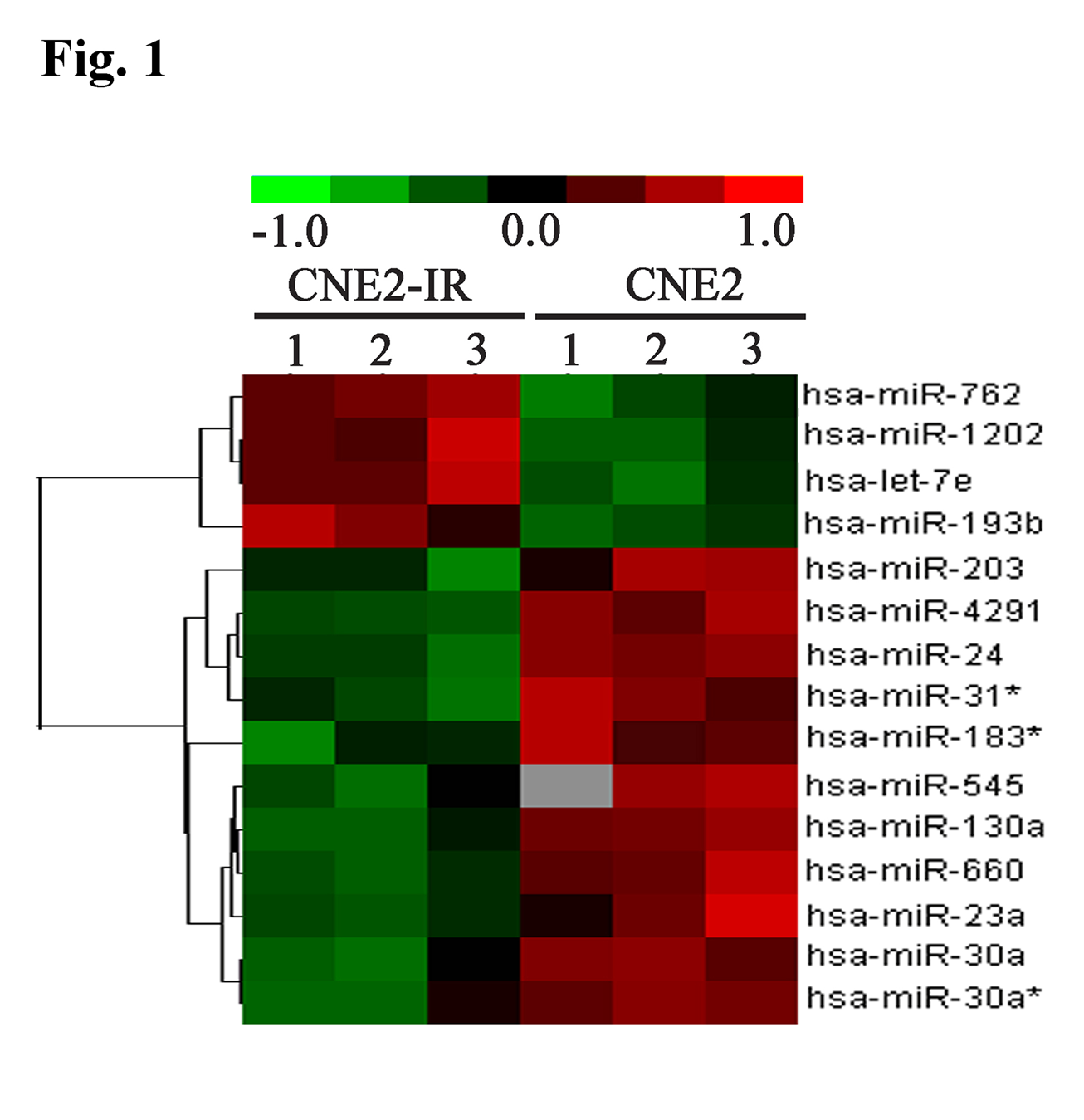

Supplement: Figure S1 — Clustering results of fifteen differentially expressed miRNAs in the CNE2-IR and CNE2 cells. Unsupervised hierarchical clustering was performed using pearson correlation coefficient and average linkage as distance and linkage metrics, respectively. Samples are well separated into CNE2-IR and CNE2 cells by the differentially expressed miRNAs. Each row represents a miRNA, and each column represents a sample. The red and green colors denote relatively high and low expression, respectively. (TIF) [file pone.0087767.s001.tif]

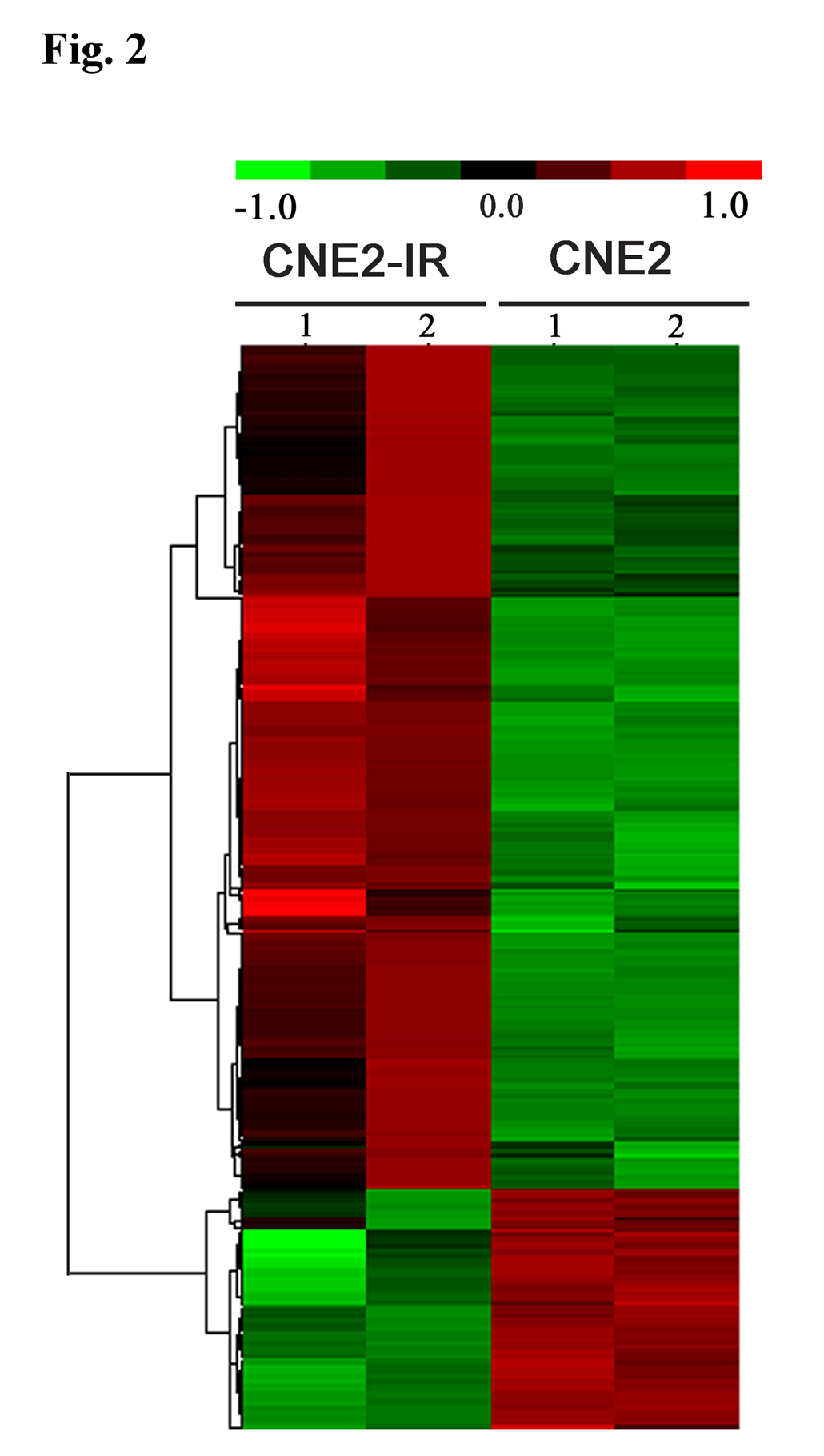

Supplement: Figure S2 — Clustering results of 372 differentially expressed mRNAs in CNE2-IR and CNE2 cells. Unsupervised hierarchical clustering was performed using pearson correlation coefficient and average linkage as distance and linkage metrics, respectively. Samples are well separated into CNE2-IR and CNE2 cells by the differentially expressed mRNAs. Each row represents a mRNA, and each column represents a sample. The red and green colors denote relatively high and low expression, respectively. (TIF) [file pone.0087767.s002.tif]
