# Supplementary material for: Integrated Analysis of Differential miRNA and mRNA Expression Profiles in Human Radioresistant and Radiosensitive Nasopharyngeal Carcinoma Cells
Source: PLoS One. 2014 Jan 31;9(1):e87767. doi: 10.1371/journal.pone.0087767 (PMC3909230; doi:10.1371/journal.pone.0087767)
Supplement: Table S1 — The clinicopathological parameters of the nasopharygeal carcinoma tissue specimens. (DOC) [file pone.0087767.s003.doc]

**Table S1. The clinicopathological parameters of the nasopharygeal carcinoma tissue specimens**

|  | | **Number** | | ***P-*value** |
| --- | --- | --- | --- | --- |
|  | **Radiosensitive NPC** | | **RadioresistantNPC** |
| **Gender**  Male | 20 | | 21 | 0.781 |
| Female | 10 | | 9 |  |
| **Age** |  | |  | 0.405 |
| ≥50 | 22 | | 19 |  |
| <50 | 8 | | 11 |  |
| **Histological type** |  | |  | 0.688 |
| WHO type II | 3 | | 4 |  |
| WHO type III | 27 | | 26 |  |
| **Primary tumor (T) stage** |  | |  | 0.718 |
| T1+ T2 | 26 | | 25 |  |
| T3+ T4 | 4 | | 5 |  |
| **Lymph node metastasis** |  | |  | 0.787 |
| Negative | 19 | | 20 |  |
| Positive | 11 | | 10 |  |
| **Clinical stage** |  | |  | 0.793 |
| II | 13 | | 12 |  |
| III | 17 | | 18 |  |
